# Supplementary material for: Accurate Chromosome Identification in the Prunus Subgenus Cerasus (Prunus pseudocerasus) and its Relatives by Oligo-FISH
Source: Int J Mol Sci. 2022 Oct 30;23(21):13213. doi: 10.3390/ijms232113213 (PMC9653872; doi:10.3390/ijms232113213)
Supplement: Supplementary file 1 [file ijms-23-13213-s001.zip › Supplement figure.pdf]

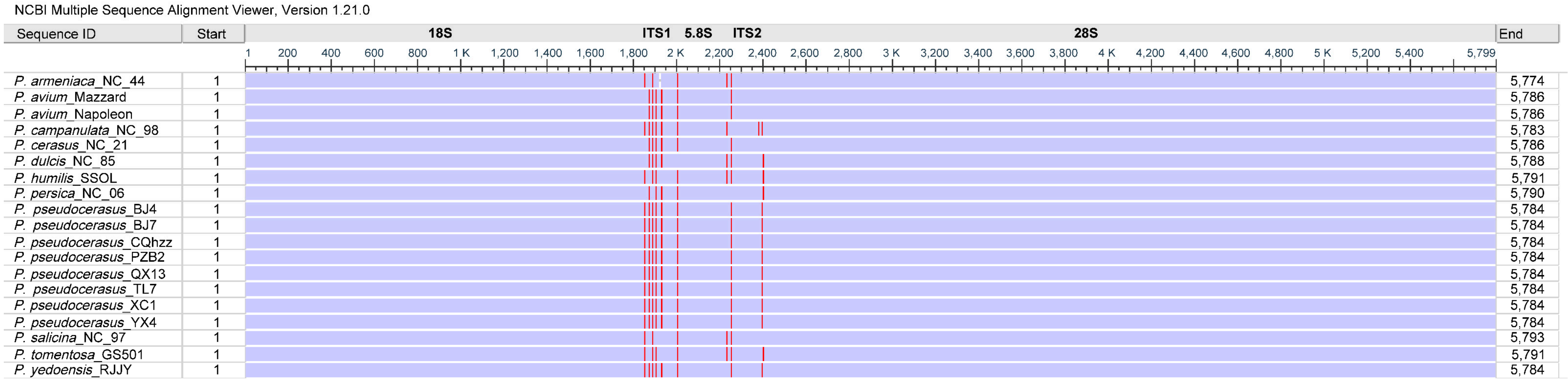

Supplementary Figure S1 The alignment of the complete 45S rDNA sequence assembled in this study.

Red lines mean the Indel positions.

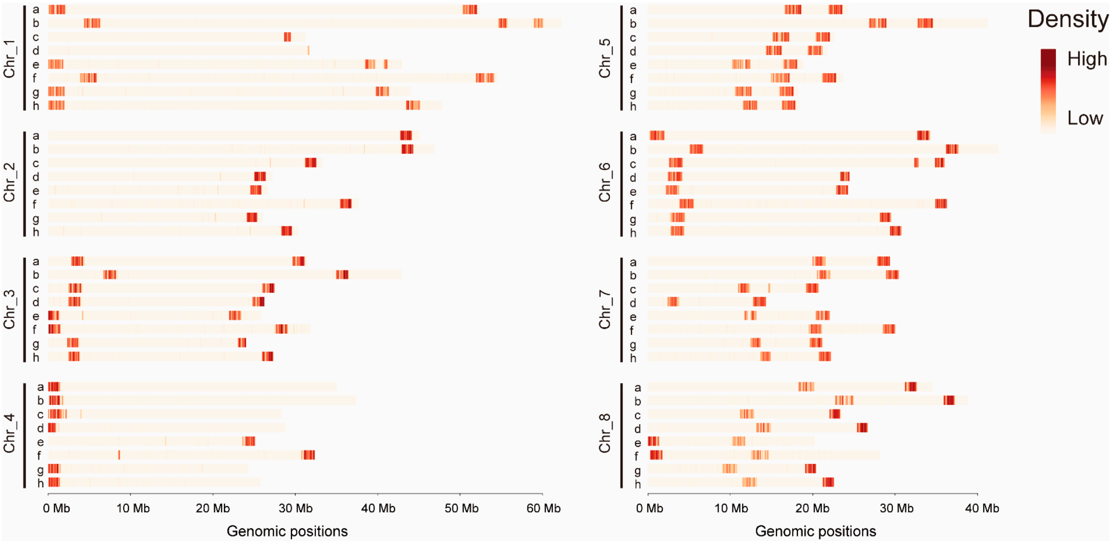

Supplementary Figure S2 Fourteen single copy oligonucleotide probe sublibraries were alignment with blast hits against the chromosome.

P. pseudocerasus (a), *P. avium* (b), *P. x yedoensis_spachiana* (c), *P. x yedoensis_speciosa* (d), *P. armeniaca* (e), *P. salicina* (f), *P. dulcis* (g), and *P. persica* (h).

Note: Chromosome renamed for *P. salicina* and *P. armeniaca* follows the draft genome of peach.

The scanning window size is 100 kb.

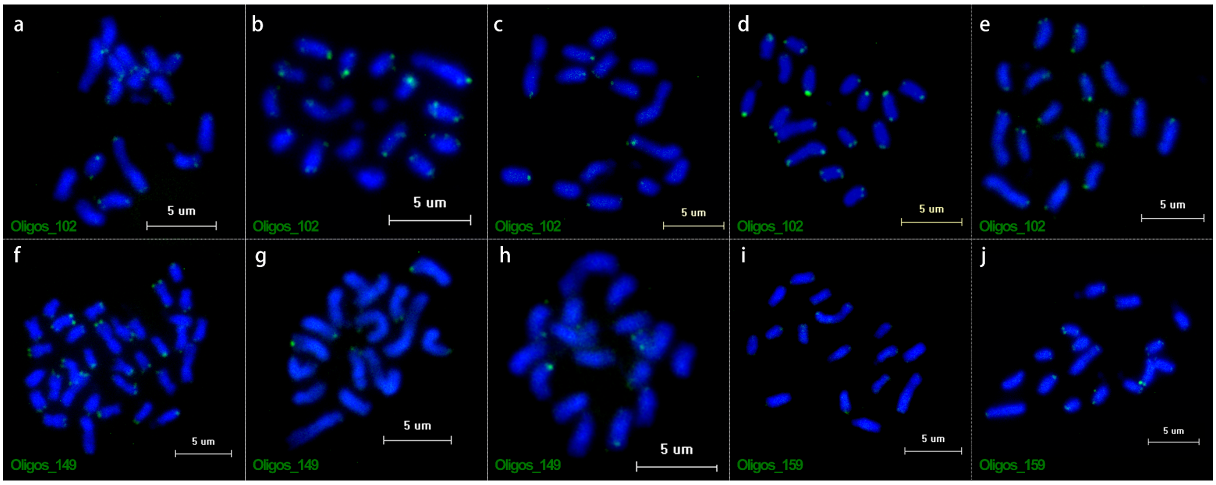

Supplementary Figure S3 The FISH of repetitive oligonucleotide probes in metaphase chromosomes of Chinese cherry and its closely related species.

(a-e) the probe distribution from monomer length of 102 bp satellite DNA sequence: *P. humilis*_Ouli (a), *P. salicina*_SYL (b), *P. armeniaca*_DGX (c), *P. dulcis*_BT (d), *P. persica*_MT (e);

(f-h) the probe distribution from monomer length of 149 bp satellite DNA sequence: *P. pseudocerasus*_HC (f), *P. campanulate*_ZHYT (g), *P. yedoensis*_DJYH (h);

(i-j) the probe distribution from monomer length of 149 bp satellite DNA sequence: *P. dulcis*_BT (i), *P. persica*_MT (j).

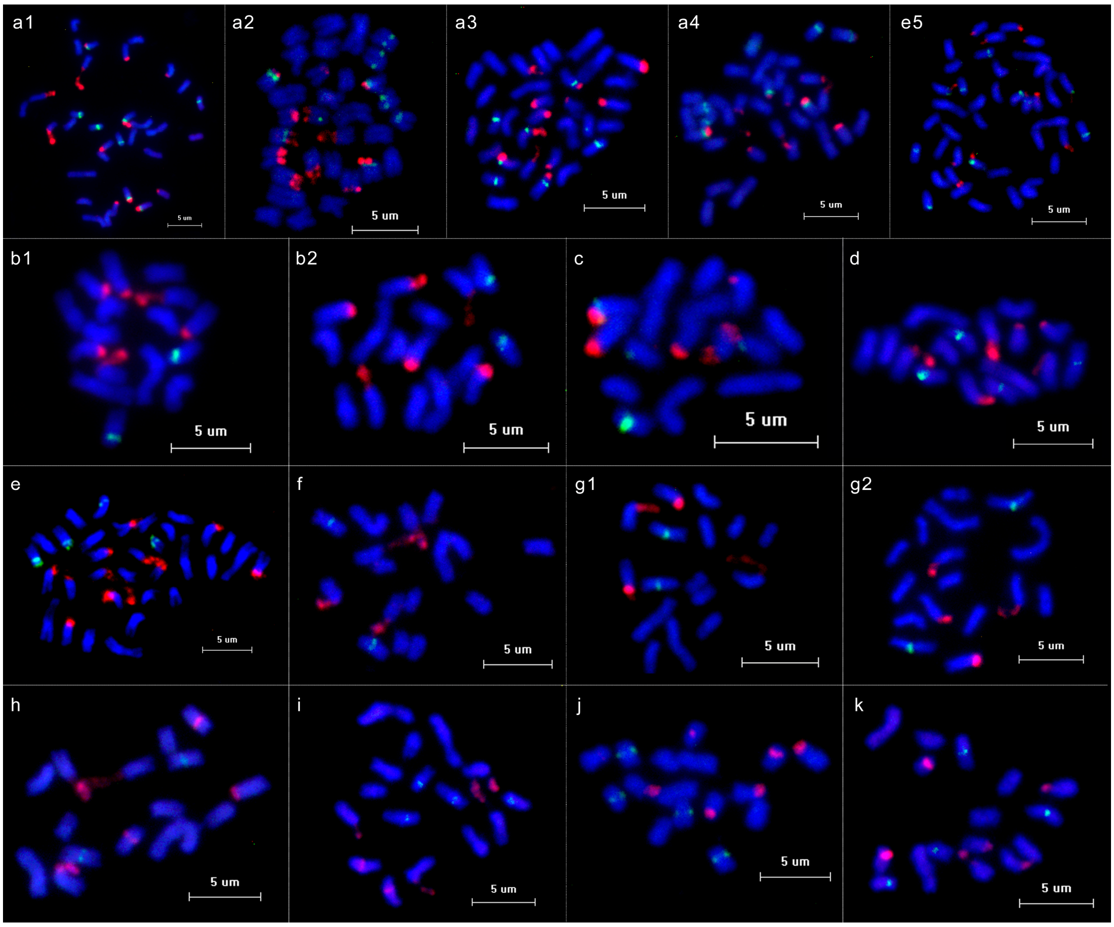

Supplementary Figure S4 The FISH of 5S/45S rDNA in metaphase chromosomes of Chinese cherry and its closely related species.

*P. pseudocerasus*_HC (a1), *P. pseudocerasus*_BZ (a2), *P. pseudocerasus*_HF (a3), *P. pseudocerasus*_PJHH (a4), *P. pseudocerasus*_FM2(a5);

*P. avium*_Mazzard (b1), *P. avium*_Van (b2), *P. yedoensis*_DJYH (c), *P. campanulate*_ZHYT (d), *P. cerasus*_SYT (e), *P. humilis*_Ouli (f), *P. tomentosa*_red_fruit (g1), *P. tomentosa*_white_fruit (g2), *P. salicina*_SYL (h), *P. armeniaca*_DGX (i), *P. dulcis*_BT (j), *P. persica*_MT (k).

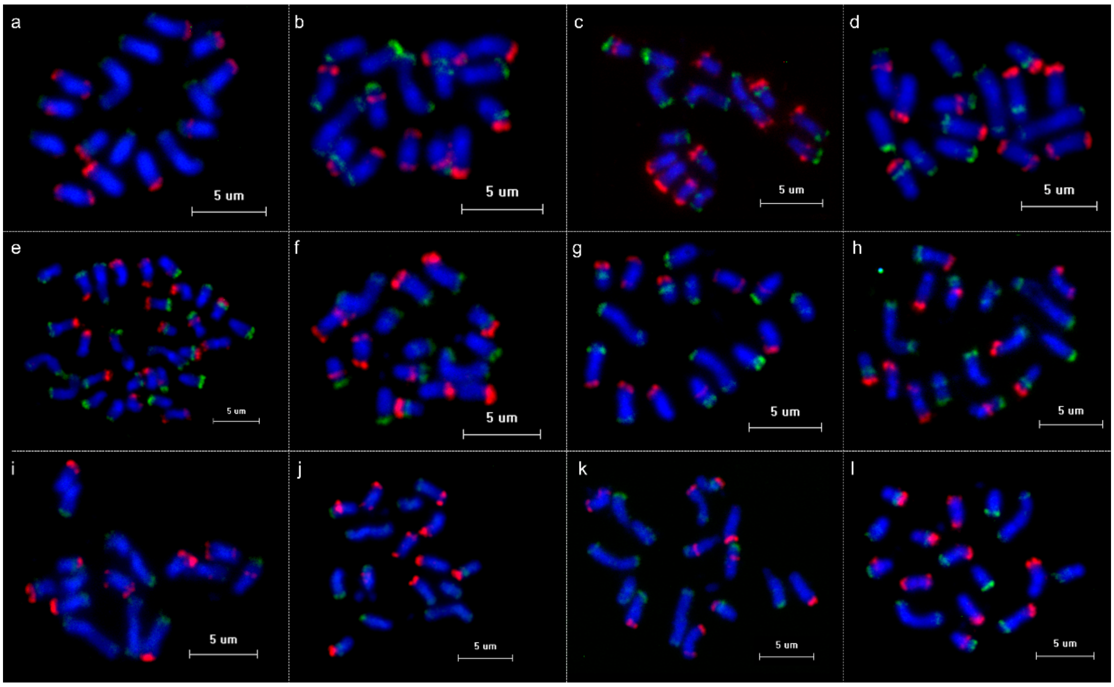

Supplementary Figure S5 Accurate chromosome identification in metaphase chromosomes from closely species related to Chinese cherry based on single copy oligonucleotide probes.

*P. avium*_Mazzard (a), *P. avium*_Van (b), *P. yedoensis*_DJYH (c), *P. campanulate*_ZHYT (d), *P. cerasus*_SYT (e), *P. humilis*_Ouli (f), *P. tomentosa*_red_fruit (g), *P. tomentosa*_white_fruit (h), *P. salicina*_SYL (i), *P. armeniaca*_DGX (j), *P. dulcis*_BT (k), *P. persica*_MT (l).

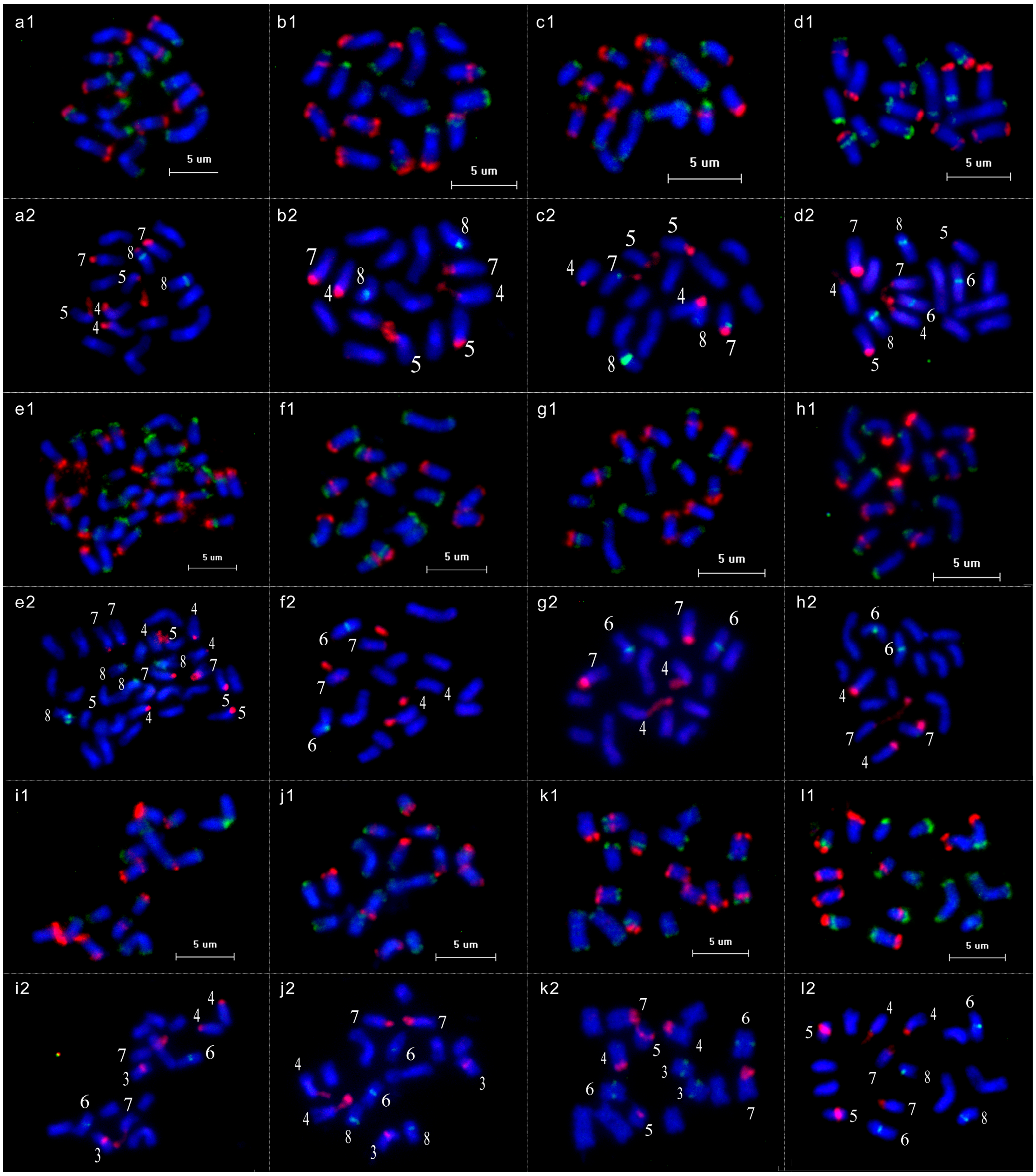

Supplementary Figure S6 The FISH of 5S/45S rDNA accurately identified metaphase chromosomes from closely species related to Chinese cherry.

(a1-l1) First round of FISH using two single copy oligonucleotide probe sublibraries on metaphase chromosomes for chromosome identification.

*Prunus avium*_Mazzard (a1), *Prunus avium*_Van (b1), *Prunus yedoensis*_DJYH (c1), *Prunus campanulate*_ZHYT (d1), *Prunus cerasus*_SYT (e1), *Prunus humilis*_Ouli (f1), *Prunus tomentosa*_red_fruit (g1), *Prunus tomentosa*_white_fruit (h1), *Prunus salicina*_SYL (i1), *Prunus armeniaca*_DGX (j1), *Prunus dulcis*_BT (k1), *Prunus persica*_MT (l1).

(a2-l2) The same cells in a1-l1 reprobed with using 5S rDNA (green), and 45S rDNA (red) probes.

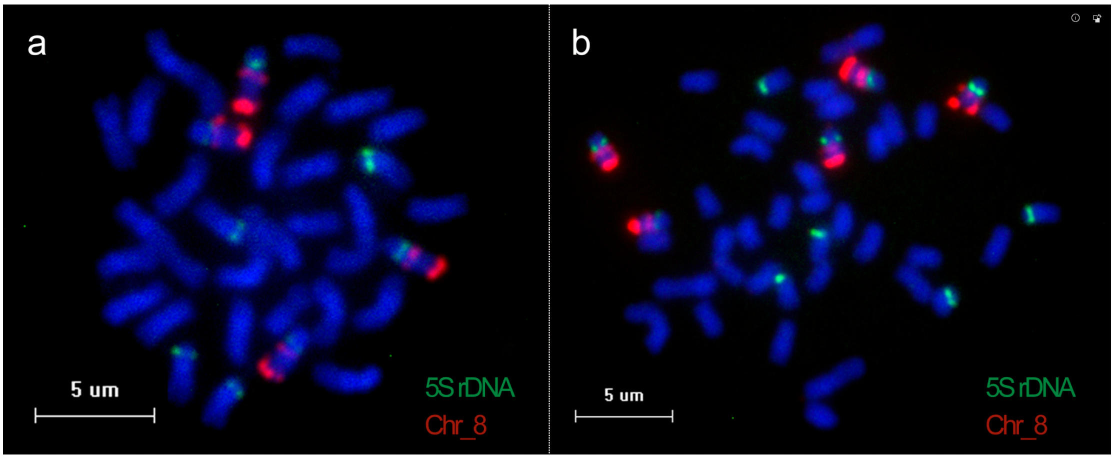

Supplementary Figure S7 The FISH of 5S rDNA and special oligonucleotide probes for Chr_8 of in metaphase chromosomes of Chinese cherry.

*P. pseudocerasus*_HC (a), *P. pseudocerasus*_FM2 (b).

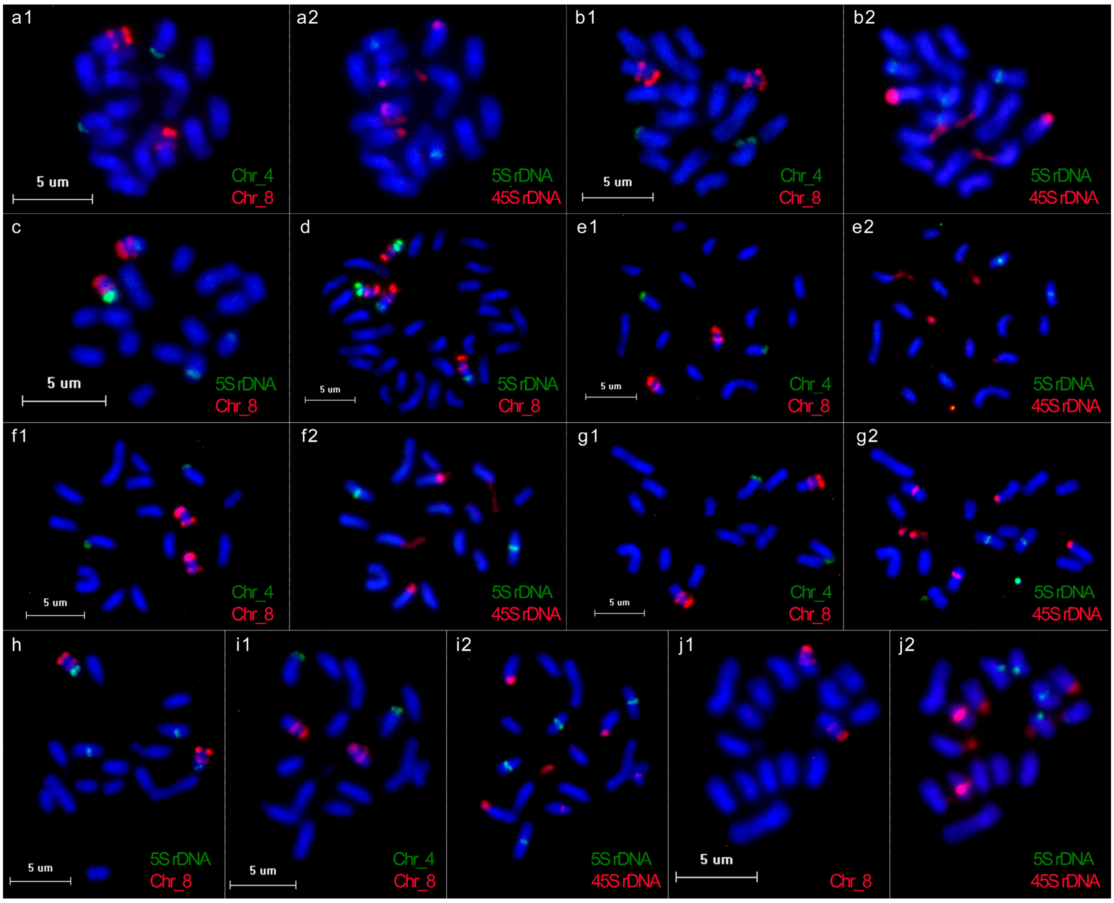

Supplementary Figure S8 The FISH of 5S/45S rDNA accurately identifies in metaphase chromosomes of closely related species to Chinese cherry based on oligonucleotide probes of Chr_4 and Chr_8.

(a1-j1) First round of FISH using two single copy oligonucleotide probe sublibraries on metaphase chromosomes for chromosome identification except for c, d, and h.

*P. avium*_Van (a1), *P. campanulate*_ZHYT (b1), *P. humilis*_Ouli (e1), *P. tomentosa*_white_fruit (f1), *P. salicina*_SYL

(g1), *P. dulcis*_BT (i1), *P. persica*_MT (j1).

(a2-j2) The same cells in a1-j1 reprobed with using 5S rDNA (green), and 45S rDNA (red) probes.

While, *P. yedoensis*_DJYH (c), *P. cerasus*_SYT (d), and *P. armeniaca*_DGX (h) with dual-colour hybridization with probes of 5S rDNA and special oligonucleotide probe sublibrary for chromosome 8.

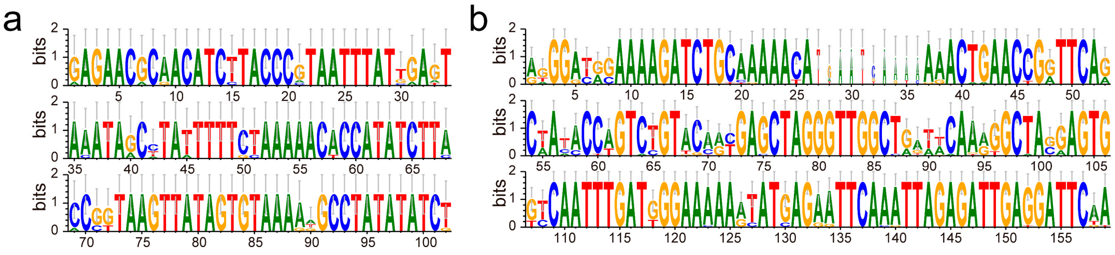

Supplementary Figure S9 Graphical representation of the consensus sequences of satellite DNA obtained by TAREAN.

The relative sizes of the letters indicate their frequency in the consensus sequence. a: the satellite DNA with the monomer length of 102 bp. b: the satellite DNA with 149 and 159 bp monomer lengths.
